# Influenza-like illness outbreaks in nursing homes in Corsica, France, 2014–2015: epidemiological and molecular characterization

**DOI:** 10.1186/s40064-016-2957-z

**Published:** 2016-08-11

**Authors:** S. Masse, L. Minodier, G. Heuze, T. Blanchon, L. Capai, A. Falchi

**Affiliations:** 1 EA 7310, Laboratory of Virology, University of Corsica-Inserm, Corte, France; 2 CIRE-SUD Paca Corse, InVS, Saint-Maurice Cedex, Paris, France; 3 UPMC Univ Paris 06, UMR_S 1136, Sorbonne Universités, Paris, France; 4 INSERM, UMR_S 1136, Paris, France

**Keywords:** Nursing homes, Influenza, Elderly, Outbreak, Influenza-like illness

## Abstract

**Background:**

To study the molecular epidemiology of the influenza outbreaks in nursing homes (NHs) to determine whether multiple influenza strains were involved.

**Methods:**

From September to December 2014, NHs in Corsica were invited to participate in an ongoing daily epidemiological and microbiological surveillance for influenza-like illness (ILI) among residents and health care workers (HCWs).

**Results:**

The study involved 12 NHs. Respiratory illness meeting the ILI case definition was observed among 44 residents from whom 22 specimens were collected. Of the 22 residents with a nasopharyngeal sample, 13 (59 %) were positive for at least one of the 11 pathogens analysed. Among these 13 patients, 11 (92 %) presented a confirmed influenza (A/H3N2) and two had another respiratory virus: one human metapneumovirus and one human coronavirus. Of patients with a confirmed influenza A(H3N2), 10 (91 %) were vaccinated against influenza during the 2014–2015 season. Two influenza outbreaks were reported in two NHs, caused by influenza A(H3N2) strains belonging to cluster 3C.3 and 3C.2a. Although antivirals were available, prophylaxis was not used.

**Conclusions:**

Phylogenetic analysis seems to suggest no multiple introduction into the two NHs reporting the two influenza A(H3N2) outbreaks. A number of factors could have contributed to transmitting influenza in NHs including, the absence of administration of antiviral treatment for prophylaxis of all residents/staff regardless of immunization status because of the poor vaccine match during each outbreak, the intensive contacts with incompletely protected residents and HCWs, and the low adherence of NHs to notification of ILI outbreaks to the health authorities.

**Electronic supplementary material:**

The online version of this article (doi:10.1186/s40064-016-2957-z) contains supplementary material, which is available to authorized users.

## Background

Elderly people, especially those with underlying comorbidities, are at risk of developing complications after infections caused by influenza viruses (Memoli et al. 2014). In developed countries, the majority of deaths attributable to influenza occur in people aged over 65 years, especially in those over 80 years with pre-existing health problems (Nicholson et al. 2003).

Outbreaks of influenza in nursing homes (NHs) are a regular occurrence during influenza epidemics (Castilla et al. 2012; Rainwater-Lovett et al. 2014; Monto et al. 2004) with influenza attack rates ranging from 1 to 65 % (Rainwater-Lovett et al. 2014) and the lethality from influenza-related complications is reported to be between 10 and 55 % (Bridges et al. 2003; Thompson et al. 2003; Gaillat et al. 2009). Routine influenza vaccination of residents and health professionals contributes to preventing these homes from being affected by waves of seasonal influenza. However, in some seasons, such in 2014–2015 when influenza vaccine effectiveness (VE) was low, especially because of a mismatch with the majority of A(H3N2) circulating viruses (Souty et al. 2015), this measure is insufficient, and more or less extensive outbreaks can occur (Nicholls et al. 2004; Monto et al. 2004). In addition to influenza vaccination and other infection-control measures, specific influenza antiviral can be administered for prophylaxis to NH residents and staff to prevent influenza outbreak (Ye et al. 2016). During the winter of 2014–2015, the antigenic drift of circulating A(H3N2) influenza viruses contributed to the decrease of influenza VE in elderly adults (Souty et al. 2015). In Europe, the 2014–2015 winter season was characterized by an excess all-cause mortality among the elderly, which was higher than during the preceding four winter seasons (Molbak et al. 2015).

To our knowledge, there have been only a few reports on the aetiology of seasonal influenza-like illness (ILI) outbreaks in NHs, but none includes a detailed molecular characterization of the influenza circulating strains (Neemuchwala et al. 2015; Mubareka et al. 2013). Here, we describe a cross-sectional study of the occurrence of 2014–2015 ILI outbreaks in 12 sentinel NHs in Corsica, France. The main objective was to study the molecular epidemiology of the influenza outbreaks in NHs to determine whether multiple influenza strains were involved in these outbreaks.

## Methods

### Nursing home recruitment and surveillance period

**Fig. 1:**
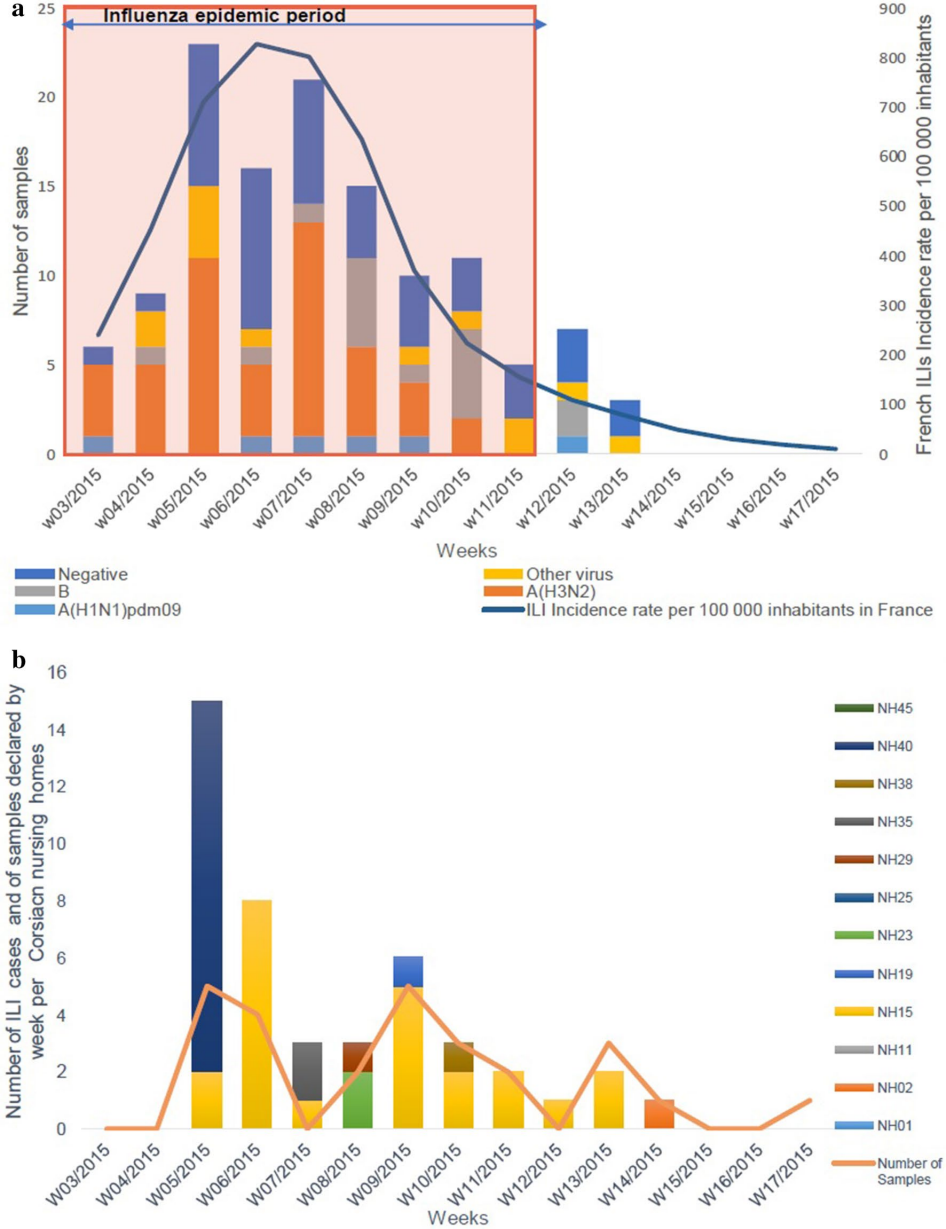
**a** Temporal distribution of respiratory viruses collected from influenza-like illness (ILI) cases consulting general practitioners of the Corsican *Sentinelles* Network between January 2015 (week 3) and April 2015 (week 17) and French ILI incidence rate per 100,000 inhabitants (*blue line*); **b** Weekly number of ILI residents declared by Nursing Homes and weekly number of ILI residents swabbed

NHs in Corsica were invited to participate in an ongoing daily epidemiological and microbiological surveillance for ILI among residents and health care workers (HCWs) including from September to December 2014. Participation was voluntary and unrestricted. Participating NHs were asked to provide data on the number and percentage of residents, and if possible, of staff vaccinated against influenza. During the 2014–2015 season, a strong influenza epidemic in metropolitan France occurred from week 3/2015 (12–18 January, 2015) to week 11/2015 (9–15 March, 2015) and peaked during week 6/2015 (2–8 February, 2015) (Fig. 1a) (van der Werf 2015), which was predominantly the result of infection by A(H3N2). Nasopharyngeal swabs were also collected by general practitioners (GPs) of the Corsican *Sentinelles* Network in a randomized sample of patients presenting with ILI (Minodier et al. 2014) (Fig. 1a).

Surveillance for ILI in NHs began at the start of influenza epidemic in week 03/2015 (12–18 January, 2015) and ended in week 17/2015 (20–26 April, 2015) (Fig. 1b).

### Case definition and surveillance

We defined a resident as a person with a registered home address in a NH. A HCW was defined as an employee, a student, and a volunteer at a NH who had regular physical contact with the residents.

At each NH, a selected HCW agreed to be an ILI surveillance officer. The HCW officer’s role was to systematically track cases among residents, and to encourage sampling of each suspected case of ILI (in either residents or HCWs).

A case of ILI was defined as a person developing within 48 h a sudden onset of any general symptoms in addition to any respiratory signs (ECDC 2015). During the ILI surveillance, the HCW officer reported weekly by phone to a research assistant the total number of residents with an ILI declared in the previous week.

Sampling was encouraged for each case of ILI. Patient information, including demographic characteristics (sex, age), symptoms, risk factors for severe influenza, treatment, 2014–2015 influenza vaccination status, and hospitalization, was documented in case report forms (CRF). The questionnaire was completed by a study nurse, who interviewed the participants and assessed the patient chart. After the interview, a nasopharyngeal swab was performed by the study nurse. The nasopharyngeal swabs and the CRF were sent daily by mail to the virology laboratory of the University of Corsica. All positive cases were followed until recovery or death.

An ILI outbreak was defined by the French Public Health Council as five cases of ILI or more clustered within 4 days among residents of a NH (HCSP 2012). NHs should report to the local health authorities as soon as an institutional outbreak defined as five cases of ILI clustered within 4 days among residents (HCSP 2012). An outbreak was considered attributable to influenza or other pathogens if at least one case was confirmed by the laboratory (Vaux et al. 2009). The duration of an outbreak was calculated using the dates of disease onset for the first and the last cases.

### Microbiological examination and phylogenetic analyses

Sample collection was performed by NHs with Σ-Virocult swabs (ELITech, France) and tested for 11 respiratory pathogens. Specimens were processed by using the duplex Respiratory Multi Well System MWS r-gene range for the following nine pathogens (Paba et al. 2014): human rhinovirus/enterovirus (HRV A-C); parainfluenza (PIV 1, 2, 3, 4); human metapneumovirus (hMPV A, B); respiratory syncytial virus (RSVA, B); human coronavirus (hCoV 229E, NL63, HKU1, OC43); adenovirus (AdV A-G); human bocavirus (hBoV 1, 2, 3, 4); *Chlamydophila pneumoniae*; and *Mycoplasma pneumoniae*. All samples were tested for influenza viruses A (A(H3N2) and A(H1N1)pdm2009) and B (Victoria and Yamagata lineage) by a real-time reverse transcription–polymerase chain reaction (RT–PCR) assay for influenza (Minodier et al. 2014).

A(H3N2) viral specimens with a cycle threshold <30 were processed and submitted to haemagglutinin (HA) sequencing. The HA sequences of A(H3N2) from seven NH residents and one NH HCW were amplified by RT–PCR using primer sets for human A(H3N2) (nucleic acids 48–1642) (Minodier et al. 2014). Twenty-one sequences detected in patients enrolled by GPs in the Corsican *Sentinelles* Network during the 2014–2015 influenza season were also included. Double-stranded sequencing of the purified PCR products (primer sequences are available on request) was performed using an Applied Biosystems Sequencer (ABI 3700, PerkinElmer). Phylogenetic trees were constructed using a neighbour-joining method based on Kimura’s two-parameter genetic distances matrix with 1000 bootstrap replicates (n = 1000) using the MEGA 6.0 program. The nucleotide sequence data from this study were deposited into GenBank at the National Center for Biotechnology Information (NCBI) (GenBank KU289606-KU289637) (http://www.ncbi.nlm.nih.gov).

### Ethics

All data were coded and tested anonymously. None of the authors collected samples. Samples and questionnaires were collected and sent to authors by the HCW at the NH involved in the surveillance. Patient information was stored according to national regulations (ethics committee ref 14-078), and access to such data was restricted. Informed consent was obtained from all participating patients. Informed consent was obtained from each patient, and the study protocol conforms to the ethical guidelines of the 1975 Declaration of Helsinki.

### Statistical analyses

Attack rates for each outbreak were calculated by dividing the total number of ILI cases among residents by the total number of residents in the NH during the outbreak. The number of residents in the entire facility was used as a denominator for the attack rate because of staff/residents mixing throughout the entire facility (e.g. common eating area for the entire facility). Vaccination coverage (VC) among NH residents was calculated by dividing the number of residents vaccinated by the total number of residents for each NH. Similarly, VC among HCWs was calculated by dividing the number of HCWs vaccinated by the total number of HCWs at each NH. Statistical analyses were performed using Stata 12.

## Results

### Characteristics of nursing homes

Among the 28 NHs solicited, 12 (43 %) agreed to provide data regarding the number of residents vaccinated against influenza (and if available, data for HCWs) and to participate in the epidemiological and microbiological surveillance of ILI. The median number of beds was 65.5 (min = 19 and max = 104). The mean size of participating homes was 62 residents and overall 92 % (min = 53 % and max = 100 %) were vaccinated against seasonal influenza for 2014–2015 (Table 1). The mean number of HCWs vaccinated against seasonal influenza for 2014–2015 was 15 % (min = 0 % and max = 47 %) (Table 1).

**Table 1:** Summary of the principal features of the 12 nursing homes (NHs) participating to the surveillance of influenza-like illness (ILIs) in Corsican, France, January–April 2015

Features	NH01	NH02	NH11	NH15	NH19	NH23	NH25	NH29	NH35	NH38	NH40	NH45	Average (min–max)
Number of residents	62	71	70	72	60	92	98	65	35	19	20	82	62 (19–98)
Number of HCWs^a^	61	47	53	52	80	53	59	36	31	12	NA^a^	70	50 (12–80)
Vaccine coverage of residents against 2014–2015 influenza seasonal vaccine	94 %	97 %	86 %	53 %	100 %	95 %	96 %	91 %	100 %	100 %	95 %	100 %	92 % (53–100)
Vaccine coverage of HCWs against 2014–2015 influenza seasonal vaccine	26 %	47 %	4 %	4 %	5 %	26 %	7 %	17 %	29 %	0 %	NA^a^	3 %	15 % (0–47)
ILIs attack rates among residents	0	1.4 %	0	32 %	2 %	2 %	0	2 %	6 %	5 %	65 %	0	9.5 % (0–65)

													N
Number of ILI^b^ residents	0	1	0	23	1	2	0	1	2	1	13	0	44
Number of ILI residents with a nasopharyngeal sample	0	1	0	13	1	1	0	0	0	0	6	0	22
Number of Influenza A(H3N2) residents	0	0	0	7	0	1	0	0	0	0	3	0	11
Other virus among residents	0	1 Human coronavirus	0	1 Human metapneumovirus	0	0	0	0	0	0	0	0	2
Number of Influenza-related hospitalisation	0	0	0	0	0	0	0	0	0	0	1	0	1
Number of Influenza-related deaths	0	0	0	2	0	0	0	0	0	0	0	0	2
Number of HCWs with a naspharyngeal sample	0	0	0	1	0	0	0	0	2	0	0	1	4
Number of Influenza A(H3N2) HCWs	0	0	0	1	0	0	0	0	0	0	0	0	1

^a^Health Care Workers

^b^Influenza-like illness

### Epidemiological and microbiological investigations

The temporal distribution of ILI cases and nasopharyngeal samples by NH are summarized in Fig. 1b. Eight (67 %) of the 12 NHs declared at least one case of ILI. Among these eight NHs, five (63 %) collected at least one nasopharyngeal sample (Table 1).

Respiratory illness meeting the ILI definition was observed in 44 residents from whom 22 specimens (50 %) were collected (Table 1). Of the 22 residents with ILI and a nasopharyngeal sample, 13 (59 %) were positive for at least one of the 11 pathogens analysed (Table 1). Among these 13 patients, 11 (92 %) presented a confirmed case of influenza (A/H3N2); one patient was positive for hMPV and one for hCov (Table 2). Of residents with a confirmed influenza A(H3N2) virus infection, 10 (91 %) were vaccinated against influenza during the 2014–2015 season. Nobody of the 22 swabbed patients received oseltamivir treatment (Table 2). Although antivirals were available, prophylaxis was not used. Three ILI outbreaks meeting the definition of an ILI cluster of five cases among residents within 4 days were documented for two NHs (NH40 and NH15) (Table 3). The first influenza outbreak was reported by NH40 in week 05/2015. This outbreak was characterized by an attack rate of 65 % (13/20), a positivity rate to influenza A(H3N2) of 50 % (3/6) and one hospitalization related to influenza A(H3N2). Droplet precautions were implemented from day one from the first case in week 05/2015. This outbreak was not declared to the French health authorities.

**Table 2:** Demographical, clinical and pathogens identified in influenza-like illness residents swabbed in nursing homes, January–April 2015

Resident characteristics	All	Influenza A(H3N2)	hMPV and hCoV	Negatif
	N = 22 (%)	N = 11 (%)	N = 2 (%)	N = 9 (%)
Age (min–max)	86.4 (72–96)	87.4 (72–96)	88.5 (83–94)	84.8 (76–93)
Gender, Female	18 (81.8 %)	10 (91 %)	1 (50 %)	7 (77.7 %)
Symptoms				
Fever >38 °C	18 (81.8 %)	10 (91 %)	2 (100 %)	6 (66.6 %)
Cough	22 (100 %)	11 (100 %)	2 (100 %)	9 (100 %)
Headache	3 (13.6 %)	1 (9.1 %)	0	2 (22.2 %)
Dyspnoea	2 (9.1 %)	1 (9.1 %)	0	1 (11.1 %)
Rhinitis	12 (54.5 %)	8 (72.7 %)	1 (50 %)	3 (33.3 %)
Asthenia	11 (50 %)	7 (63.6 %)	0	4 (44.4 %)
Conjunctival hyperemia	2 (9.1 %)	2 (18.2 %)	0	0
Other	6 (27.3 %)	4 (36.4 %)	0	2 (22.2 %)
Seasonal influenza vaccination 2014–2015	21 (95.5 %)	10 (91.0 %)	2 (100 %)	9 (100 %)
Chronic disease	17 (77.2 %)	8 (72.7 %)	2 (100 %)	7 (77.7 %)
Oseltamivir treatement	0	0	0	0
Antibiotic treatement	8 (36.4 %)	4 (36.4 %)	2 (100 %)	2 (22.2 %)
Influenza-related hospitalization	1 (4.5 %)	1 (9 %)	0	0
Influenza- related death	2 (9 %)	2 (18.2 %)	0	0

**Table 3:** Influenza- like illness outbreaks in NH40 and in NH15

	NH40	NH15
Number of residents (N)	20	72
Number of HCWs^a^ (N)	NA	52
Residents influenza vaccination coverage 2014–2015 % (N)	95 % (19)	53 % (38)
HCWs influenza vaccination coverage 2014–2015 % (N)	NA	3.8 % (2)

	Outbreak 1	Outbreak 2	Outbreak 3
Week(s) of outbreak	w05/2015	w06/2015	w09/2015–w13/2015
Attack rate among residents % (N)	65 % (13/20)	11 % (8/72)	16.6 % (12/72)
Number of ILI^b^ residents with a nasopharyngeal sample	6	4	9
ILI residents positives to at least one pathogen % (N)	50 % (3/6)	25 % (1/4)	78 % (7/9)
Pathogen detected in residents	A(H3N2)	human metapneumovirus	A(H3N2)
Number of influenza-related hospitalization	1	0	0
Number of influenza- related death	0	0	2
Number of HCWs with a nasopharyngeal sample	0	0	1
HCW with influenza A(H3N2)	0	0	1

*NA* not available

^a^Health care workers

^b^Influenza-like illness

The second outbreak was declared by NH15 in week 6 (Table 3), with an attack rate of 11 % (8/72). One of the four swabbed residents was positive for hMPV (25 %). The third outbreak was declared by NH15, starting in week 09/2015 and ending in week 13/2015 (Table 3). The attack rate was 16.6 % (12/72) with a positivity rate to influenza A(H3N2) of 78 % (7/9). One HCW with ILI at NH15 was swabbed and a positive result for A(H3N2) infection was found in week 9/2015. Two residents of NH15 who were positive for A(H3N2) died in weeks 9/2015 and 11/2015. This outbreak was declared to the French health authorities in week 10/2015 (3 March, 2015). Droplet precautions were implemented from day one of the first case in week 09/2015. NH15 did not require advice from the public health authorities for outbreak management.

### Molecular investigation of the two influenza outbreaks

**Fig. 2:**
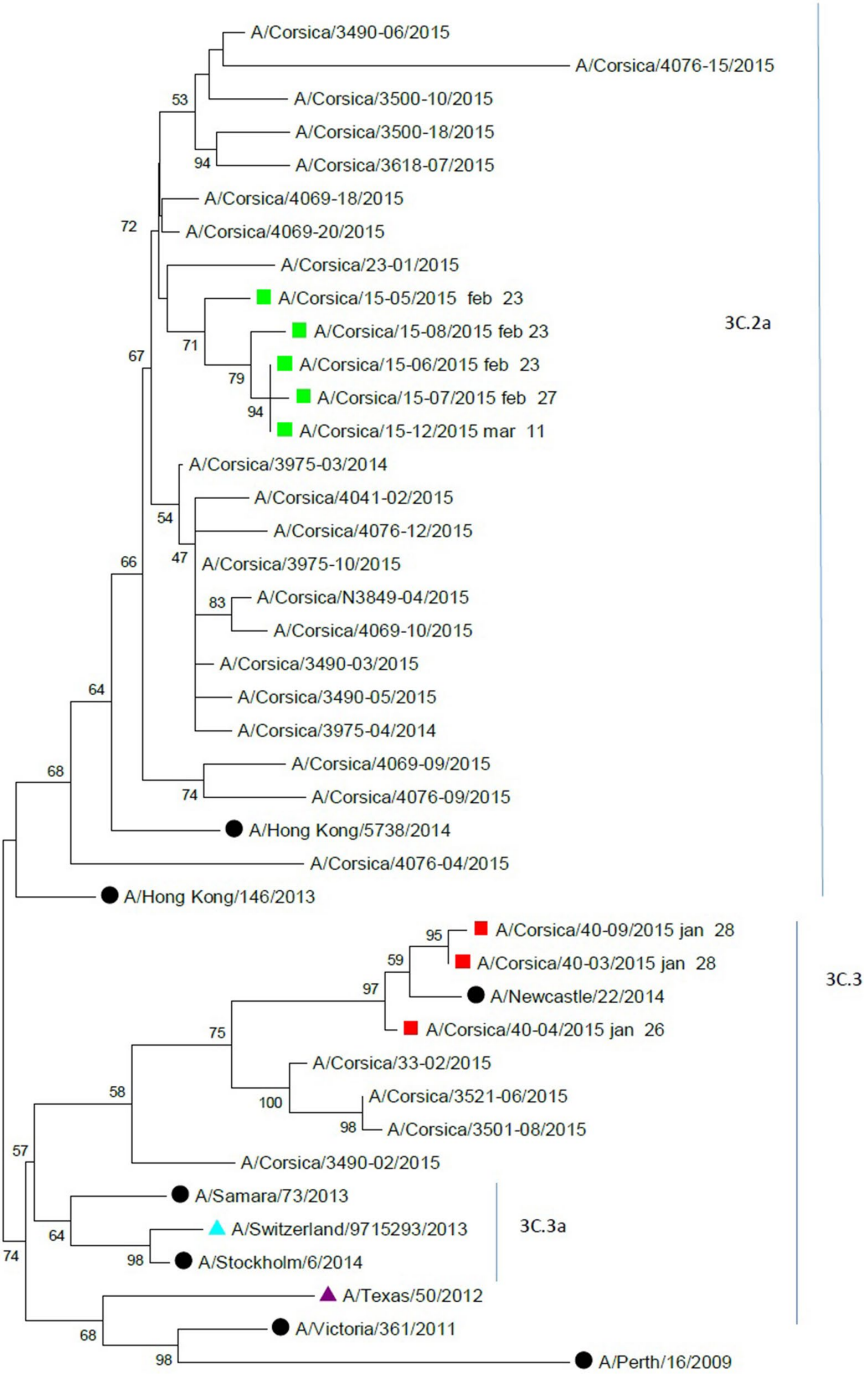
Phylogenetic analysis of hemagglutinin (HA) nucleotide sequences of influenza A(H3N2). Influenza A(H3N2) strains detected in NH15 and NH40 are indicated by *red* and *green squares* respectively. Vaccine strains of the 2014–2015 season and of the 2015–2016 seasons are indicated by *triangles* and the other vaccine/reference strains by *circles*. Phylogenetic trees were constructed using the MEGA 6.0 program (Tamura et al. 2013)

The characteristics of the seven residents and single HCW with influenza A(H3N2) belonging to NH15 and NH40 included in the phylogenetic analysis are summarized in Additional file 1. Twenty-one HA sequences detected in A(H3N2) patients enrolled by GPs of the Corsican *Sentinelles* Network during the 2014–2015 influenza season were also included. Phylogenetic analysis of the HA of A(H3N2) viruses was performed (Fig. 2 and Additional file 2).

All strains were characterized by the mutation N145S. The HA of NH15 clustered in the A/Hong Kong/5738/2014-like group 3C.2a. The group 3C.2a strains were characterized by the mutations N144S (antigenic site A), F159Y (antigenic site B), N225D and Q311H (antigenic site C) with respect to A/Texas/50/2012 (reference vaccine strain for 2014–2015). The HA of the A(H3N2) virus, which infected the HCW (A/Corsica/15-08/2015) was the same as the HA detected in residents of NH15 (Fig. 2 and Additional file 2).

The HA of NH40 clustered in the A/Newcastle/22/2014-like group 3C.3. The group 3C.3 strains were characterized by mutations E62 K, K83R, and R261Q (antigenic site E), N122D (antigenic site A), and L157S (antigenic site B) with respect to A/Texas/50/2012 (reference vaccine strain for 2014–2015) (Fig. 2 and Additional file 2).

## Discussion

Phylogenetic analysis suggests no multiple introduction of influenza A(H3N2) strains into NHs as the two influenza outbreaks investigated were caused by influenza viruses belonging to clades 3C.3 and 3C.2a respectively. Several concomitant factors could all have contributed to transmitting influenza in NHs, including (1) the absence of administration of antiviral treatment for prophylaxis of all residents/staff regardless of immunization status because of the poor vaccine match during each outbreak, (2) the intensive contacts with incompletely protected residents and HCWs and (3) the low adherence of NHs to notification of ILI outbreaks to the health authorities.

In this study, more than 90 % of confirmed cases of influenza A(H3N2) infection involved in the NH influenza outbreaks were in patients vaccinated against 2014–2015 seasonal influenza. These results are consistent with the low rate of VE in ≥65 year-olds reported in general practice in France, which was the lowest influenza VE estimate for the four previous seasonal influenza epidemics. The phylogenetic tree analysis of HA sequences showed that the A(H3N2) influenza virus strains that caused the outbreaks in NH15 and NH40 residents belonged to two distinct clusters, 3C.2a and 3C.3, respectively. Both of these clades together were found in over 70 % of influenza A(H3N2) viruses for which phylogenetic groups were determined (ECDC 2015). These two clusters are antigenically distinct from the A/Texas/50/2012 (influenza vaccine strain for 2014–2015), and the WHO has recommended that the A(H3N2) component should be updated with an A/Switzerland/9715293/2013-like (HA 3C.3a) virus for the Northern Hemisphere 2015–2016 vaccine (Blanchon et al. 2015). The HA of A(H3N2) strains detected in NH15 belonged to the cluster 3C.2a, which brings together the majority of influenza strains detected in general medicine in Corsica during the 2014–2015 season.

The HCW (A/Corsica/15-08/2015) might have had the primary case, having the same strain as residents of NH15. The transmission of influenza from HCWs to patients is very common and has been described for other nosocomial influenza outbreaks (Oguma et al. 2011; Eibach et al. 2014). Other factors in NHs could have supported the spread of influenza outbreaks including the older age of residents, the high prevalence of multiple chronic disease, family visits, communal living arrangements, shared caregiving, the continual close proximity of residents and ineffectual infection-control programs (Montoya et al. 2016). Overall these factors could have facilitate both the introduction and subsequent transmission of influenza viruses among residents of the NH15 and NH40. When an influenza outbreak is suspected in an institutionalized setting, it is recommended to intensify measures to avoid transmission such as frequent hand washing, use of face masks, separation of sick persons from the rest of the residents, reducing visits and reducing staff movement between different areas of the NH (HCSP 2012). Antiviral drug treatment in cases and in persons exposed may also be useful (HCSP 2012). Notably no influenza antiviral treatment had been administrated in NHs included in our study for treatment and/or prophylaxis of residents/staff. These results are an agreement with a previous study describing the absence of antiviral treatment for prophylaxis during NHs influenza outbreaks in Spain (Castilla et al. 2012) and with the low rate of neuraminidase inhibitors prescription to ILI patients with a severe influenza risk factor reported in French primary health care (Blanchon et al. 2015).

Seasonal influenza vaccination of NH residents and of HCWs and the early outbreak notification are important control measures in NHs. If the impact of influenza vaccination campaign is demonstrated by the high VC of NH residents included in this study (92 vs. 48.5 % in the general population for the community-dwelling elderly) (INPES 2015) influenza vaccine uptake among HCWs was insufficient (15 %; min = 0 and max = 47) (WHO 2003). The low level of VC among HCWs was in agreement with previous studies, ranging from <10 to 50 % (Vaux et al. 2010). All NHS included in this study conducted surveillance of ILI routinely. In this study, only one ILI outbreak (detected in NH15) was declared to the health authorities. This suggests that NHs still encounter difficulties in reporting surveillance data and that the actual system based on report outbreaks to the local health authorities, need to be implemented.

This study has several limitations. First, the actual number of ILI and the actual extent of the outbreaks may have been under-estimated because residents or HCWs who were mildly infected or asymptomatic were not included. Furthermore the ILI outbreak definition was not sensitive enough and other outbreaks could have been missed. Second, some requested that data should not be available for all NH participants, such as HCW influenza VC. Third, even if the detection of respiratory viruses using RT–PCR is a highly sensitive method, there is a potential bias for detection as the viral load in samples from elderly adults is generally lower than that in samples from younger adults (Lee et al. 2009). On the other hand the duration of shedding is higher in the elderly, so that may facilitate the identification of causative organisms (Talbot and Falsey 2010). Fourth, visitors occasionally passing through the NHs were not monitored for ILI because of logistical barriers.

The strengths of this study design were the supervision of epidemiological and microbiological data in NHs and the usefulness of molecular typing techniques to elucidate the epidemiology of influenza cross-contamination.

In summary, the current study provides further evidence of the impact of influenza outbreaks in NHs and that genotyping is an excellent tool to further our understanding of transmission dynamics. Influenza is a serious problem for NHs, and the current study highlights the need to improve effective management of future influenza outbreaks.

## Additional files

10.1186/s40064-016-2957-z Characteristic of residents and health care workers with A(H3N2) included in phylogenetic analysis.
10.1186/s40064-016-2957-z Amino acid substitutions observed in antigenic sites (A-E) of the hemagglutinin protein of eight A(H3N2) influenza viruses detected in residents and health care workers in nursing homes, Corsica, 2014–2015.
