# Supplementary material for: Influenza-like illness outbreaks in nursing homes in Corsica, France, 2014–2015: epidemiological and molecular characterization
Source: Springerplus. 2016 Aug 11;5(1):1338. doi: 10.1186/s40064-016-2957-z (PMC4981007; doi:10.1186/s40064-016-2957-z)
Supplement: Supplementary file 1 — 10.1186/s40064-016-2957-z Characteristic of residents and health care workers with A(H3N2) included in phylogenetic analysis. [file 40064_2016_2957_MOESM1_ESM.docx]

**Influenza-like illness outbreaks in nursing homes in Corsica, France, 2014–2015: epidemiological and molecular characterization.**

S. Masse ^1^, L. Minodier ^1^, G. Heuze ^2^, T. Blanchon^3,4^, L. Capai ^1,3,4^, A. Falchi^1^*

^1^EA 7310, laboratory of virology, University of Corsica-Inserm, Corte, France

^2^CIRE-SUD Paca Corse, InVS

^3^Sorbonne Universités, UPMC Univ Paris 06, UMR_S 1136, Paris, France.

^4^INSERM, UMR_S 1136, Paris, France

*Corresponding author: Alessandra Falchi, PhD

EA 7310, Laboratory of Virology,

University of Corsica-Inserm,

20250 Corte, France,

Mail : [falchi@univ-corse.fr](mailto:falchi@univ-corse.fr), Telephone: +0033495450677

**Additional file 1**: Characteristic of residents and health care workers with A(H3N2) included in phylogenetic analysis

| **Case no** | **Sample** | **Age(years)** | **Gender** | **Influenza 2014-2015 vaccination status** | **Date of sample** | **Week** | **Hospitalization** | **Death** | **Sequences** |
| --- | --- | --- | --- | --- | --- | --- | --- | --- | --- |
| 15-05 | Resident | 77 | F | YES | 23/02/2015 | 9 | NO | NO | YES |
| 15-06 | Resident | 90 | F | YES | 23/02/2015 | 9 | NO | NO | YES |
| 15-07 | Resident | 87 | M | NO | 27/02/2015 | 9 | NO | YES | YES |
| 15-08 | HCW* | 25 | F | NO | 23/02/2015 | 9 | NO | NO | YES |
| 15-09 | Resident | 96 | F | YES | 23/02/2015 | 13 | NO | NO | NON |
| 15-11 | Resident | 94 | F | YES | 12/03/2015 | 11 | NO | YES | NON |
| 15-12 | Resident | 90 | F | YES | 11/03/2015 | 11 | NO | NO | YES |
| 40-03 | Resident | 86 | F | YES | 28/01/2015 | 5 | YES | NO | YES |
| 40-04 | Resident | 93 | F | YES | 26/01/2015 | 5 | NO | NO | YES |
| 40-09 | Resident | 84 | F | YES | 28/01/2015 | 5 | NO | NO | YES |

*HCW: Health care worker
