# Supplementary material for: Influenza-like illness outbreaks in nursing homes in Corsica, France, 2014–2015: epidemiological and molecular characterization
Source: Springerplus. 2016 Aug 11;5(1):1338. doi: 10.1186/s40064-016-2957-z (PMC4981007; doi:10.1186/s40064-016-2957-z)
Supplement: Supplementary file 2 — 10.1186/s40064-016-2957-z Amino acid substitutions observed in antigenic sites (A-E) of the hemagglutinin protein of eight A(H3N2) influenza viruses detected in residents and health care workers in nursing homes, Corsica, 2014–2015. [file 40064_2016_2957_MOESM2_ESM.docx]

**Influenza-like illness outbreaks in nursing homes in Corsica, France, 2014–2015: epidemiological and molecular characterization.**

S. Masse ^1^, L. Minodier ^1^, G. Heuze ^2^, T. Blanchon^3,4^, L. Capai ^1,3,4^, A. Falchi^1^*

^1^EA 7310, laboratory of virology, University of Corsica-Inserm, Corte, France

^2^CIRE-SUD Paca Corse, InVS

^3^Sorbonne Universités, UPMC Univ Paris 06, UMR_S 1136, Paris, France.

^4^INSERM, UMR_S 1136, Paris, France

*Corresponding author: Alessandra Falchi, PhD

EA 7310, Laboratory of Virology,

University of Corsica-Inserm,

20250 Corte, France,

Mail : [falchi@univ-corse.fr](mailto:falchi@univ-corse.fr), Telephone: +0033495450677

**Additional file 2**: Amino acid substitutions observed in antigenic sites (A-E) of the hemagglutinin protein of eight A(H3N2) influenza viruses detected in residents and health care workers in nursing homes, Corsica, 2014-2015.

| **Epitopes** | **E** | **E** | **A** | **B** | **A** | **A** | **A** | **A** | **B** | **B** | **B** | **A** | **B** | **B** | **B** | **C** | **E** | **C** |  |
| --- | --- | --- | --- | --- | --- | --- | --- | --- | --- | --- | --- | --- | --- | --- | --- | --- | --- | --- | --- |
| **Antigenic sites** | **62** | **83** | **122** | **128** | **138** | **142** | **144** | **145** | **157** | **159** | **160** | **168** | **186** | **197** | **198** | **225** | **261** | **311** | **347** |
| A/Texas/50/2012 | E | K | N | N | A | R | N | N | L | F | K | M | G | Q | P | N | R | Q | V |
| A/Hong Kong/146/2013 | . | . | . | T | . | . | . | S | . | . | . | . | . | . | S | . | . | . | . |
| A/Hong Kong/5738/2014 | . | . | . | T | . | . | S | S | . | Y | . | . | . | . | S | D | . | H | . |
| A/Corsica/15-05/2015 | . | . | . | T | . | . | S | S | . | Y | T | . | . | . | S | D | . | H | . |
| A/Corsica/15-06/2015 | . | . | . | T | . | . | S | S | . | Y | T | V | . | . | S | D | . | H | . |
| A/Corsica/15-07/2015 | . | . | . | T | . | . | S | S | . | Y | T | V | . | . | S | D | . | H | . |
| A/Corsica/15-08/2015* | . | . | . | T | . | . | S | S | . | Y | T | V |  | . | S | D | . | H | . |
| A/Corsica/15-12/2015 | . | . | . | T | . | . | S | S | . | Y | T | V | . | . | S | D | . | H | . |
| A/Samara/73/2013 | . | . | . | A | . | G | . | S | . | . | . | . | . | . | S | . | . | . | . |
| A/Switzerland/9715293/2013 | . | . | . | A | S | G | . | S | . | S | . | . | V | . | S | D | . | . | . |
| A/Stockholm/6/2014 | . | . | . | A | S | G | . | S | . | S | . | . | . | . | S | D | . | . | . |
| A/Newcastle/22/2014 | K | R | D | A | S | G | . | S | S | . | . | . | . | . | S | . | Q | . | K |
| A/Corsica/40-03/2015 | K | R | D | A | . | G | . | S | S | . | . | . | . | . | S | . | Q | . | K |
| A/Corsica/40-04/2015 | K | R | D | A | . | G | . | S | S | . | . | . | . | . | S | . | Q | . | . |
| A/Corsica/40-09/2015 | K | R | D | A | . | G | . | S | S | . | . | . | . | . | S | . | Q | . | K |

* Health-care worker
